# Septic shock due to *Escherichia coli* meningoencephalitis treated with immunoglobulin-M-enriched immunoglobulin preparation as adjuvant therapy: a case report

**DOI:** 10.1186/s13256-021-02731-7

**Published:** 2021-03-29

**Authors:** V. Pota, M. B. Passavanti, F. Coppolino, F. Di Zazzo, L. De Nardis, R. Esposito, M. Fiore, G. S. R. C. Mangoni di Santostefano, C. Aurilio, P. Sansone, M. C. Pace

**Affiliations:** Dept of Women, Child, General and Specialist Surgery, University of Campania “L. Vanvitelli”, Naples, Italy

**Keywords:** Sepsis, Septic shock, Enriched IgM immunoglobulin preparation, *Escherichia coli*, Meningoencephalitis

## Abstract

**Background:**

Gram-negative bacteria are an uncommon etiology of spontaneous community-acquired adult meningitis and meningoencephalitis. *Escherichia coli* is a Gram-negative bacterium that is normally present in the intestinal microbial pool. Some *Escherichia coli* strains can cause diseases in humans and animals, with both intestinal and extraintestinal manifestations (extraintestinal pathogenic *Escherichia coli*) such as urinary tract infections, bacteremia with sepsis, and, more rarely, meningitis. Meningitis continues to be an important cause of mortality throughout the world, despite progress in antimicrobial chemotherapy and supportive therapy. The mortality rate fluctuates between 15% and 40%, and about 50% of the survivors report neurological sequelae. The majority of *Escherichia coli* meningitis cases develop as a result of hematogenous spread, with higher degrees of bacteremia also being related to worse prognosis. Cases presenting with impaired consciousness (that is, coma) are also reported to have poorer outcomes.

**Case presentation:**

We describe the case of a 48-year-old caucasian woman with meningoencephalitis, with a marked alteration of consciousness on admission, and septic shock secondary to pyelonephritis caused by *Escherichia coli*, treated with targeted antimicrobial therapy and immunoglobulin-M-enriched immunoglobulin (Pentaglobin) preparation as adjuvant therapy.

**Conclusion:**

Despite the dramatic presentation of the patient on admission, the conflicting data on the use of immunoglobulins in septic shock, and the lack of evidence regarding their use in adult *Escherichia coli* meningoencephalitis, we obtained a remarkable improvement of her clinical condition, accompanied by partial resolution of her neurological deficits.

## Background

Bacterial meningitis is a severe and life-threatening infectious disease, and represents a relevant cause of mortality throughout the world, despite advances in antimicrobial and adjuvant therapy, supportive care, and epidemiological prevention strategies [1, 2]. Overall estimated incidence in Western countries is two to six cases per 100,000 people each year, being likely much higher in less-developed areas [1, 2]. The rate of neurological sequelae and disability among survivors is reported to be around 30–50% [1–3]. Gram-negative bacteria are an uncommon etiology of spontaneous community-acquired meningitis in adults, representing only 0.7–8.7% of adult meningitis cases according to different authors [4, 5]. Among Gram-negative bacteria, *Escherichia coli* (*E. coli*) represents 41.9% of all community-acquired Gram-negative cases in adults, accounting for an overall percentage of community-acquired meningitis in adult patients of about 1–3% [5–8].

## Case presentation

A 48-year-old caucasian woman (weighing 60 kg) was transferred from the emergency room (ER) of Boscotrecase (Naples, Italy) to the intensive care unit (ICU) of University of Campania “L. Vanvitelli” for suspected pyelonephritis with systemic impairment, fever, sepsis, and altered mental state (coma). She had no history of relevant comorbidities or particular risk factors such as immunosuppression.

In the ER, she underwent a computerized tomography (CT) scan of abdomen, chest, and brain with and without contrast enhancement. The CT scan demonstrated “moderate ectasia of the right renal calyx with peripheral medullar densitometric alterations,” being suggestive for pyelonephritis. On admission in our ICU, the patient was sedated, intubated with an orotracheal tube, monitored, and ventilated in a controlled mode. Vital signs on admission were arterial pressure 80/40 mmHg [mean arterial pressure (MAP), 53 mmHg]; heart rate 110 beats per minute (bpm), peripheral saturation of O_2_ (SpO_2_) 100%. Her body temperature was 39 °C; lactate values were 6.2 mmol/l. Right after admission, the patient’s monitoring was switched from noninvasive to invasive, with cannulation of the left radial artery and monitoring of hemodynamic parameters with the Vigileo system (a device that analyzes arterial blood pressure waveforms and their variations).

The hemodynamic parameters monitored with Vigileo showed cardiac output (CO) 2.1 l/minute (normal range 4.0–8.0 l/minute), systemic vascular resistance (SVR) 350 dyne seconds/cm^5^ (normal range 800–1200 dyne seconds/cm^5^) (MAP 53 mmHg).

Routine blood tests were performed, in addition to procalcitonin (PCT) sampling, serology for hepatotrophic viruses and human immunodeficiency virus (HIV), and a multiplex polymerase chain reaction (PCR) molecular biological blood sampling for detection of nucleic acids of bacteria, viruses, and fungi. Urine routine analysis along with microbiological tests was performed as well. A brief sedation window was performed, and neurological examination demonstrated a coma state with a Glasgow Coma Scale (GCS) score of 5 (Eye 1, Vocal 1T, Motor 3), with a decorticated response to pain, bilaterally myotic pupils with a torpid pupillary response, and a bilaterally positive Babinski sign.

Early fluid resuscitation began with a bolus of 30 ml/kg of crystalloid in 3 h, and norepinephrine infusion began at a rate of 0.2 µg/kg/minute [9, 10].

Empirical antibiotic therapy with ceftazole/tazobactam (1 g/0.5 g every 8 hours), meropenem (1 g every 8 hours), and aciclovir (250 mg) was administered. Dexamethasone was added as adjuvant therapy (10 mg once per day for 4 days) [1].

Blood samples revealed white blood cells (WBC) 11.00 × 10^3^/μl (normal range 4.2–9.0 × 10^3^/µl) (neutrophils 86.0%, lymphocytes 12.6%), red blood cells (RBC) 3.97 × 10^6^/μl (normal range 4.5–6.1 × 10^6^/µl), hemoglobin (HB) 10.2 g/dl (normal range for women 12–16 g/dl), platelets (PLT) 54 × 10^3^/μl (normal range 150–450 × 10^3^/μl), procalcitonin (PCT) 61 ng/ml (normal range < 0.5 ng/ml), and C-reactive protein (CPR) 17.5 mg/dl (normal range < 0.5 mg/dl).

Furthermore, blood PCR analysis was positive for *E. coli*. The analysis was negative for *N. meningitidis*, *H. influenzae*, and *S. pneumoniae*.

Urine microbiological examination was also positive for *E. coli*, with a total microbial load (colony-forming units, CFU) of 10,000 CFU/ml. The antibiogram showed high sensitivity of *E. coli* to meropenem. We therefore decided to suspend ceftazole/tazobactam and continue therapy with meropenem.

After etiologic diagnosis, in consideration of the septic shock condition and the relatively young age of the patient, it was decided to introduce an immunoglobilins (Ig)M-enriched intravenous immunoglobulin (IVIG) preparation (Pentaglobin^®^) at a dosage of 250 ml/kg per day for 4 days. Pentaglobin^®^ (immunoglobulin IgM-enriched; Biotest) is a plasma-derived solution with the following composition: 12% IgM, 76% IgG, 12% IgA. Although the Surviving Sepsis Campaign guidelines advise against the use of IVIG in patients with sepsis or septic shock, given the lack of a statistical significance for survival benefits [9, 10], our recent positive outcome in treating septic shock with an IgM-enriched formulation as an adjuvant therapy [11] and the Gram-negative etiology of the patient’s condition were a convincing rationale, as IgM-enriched IVIGs were found to have higher antibody levels against *Escherichia* *coli* and other Gram-negative bacteria than did normal IVIG preparations [12].

After 24 hours of therapy, the patient showed an improvement in blood chemistry (CPR 8.3 mg/dl; PCT 7.7 ng/ml; lactate 4.1 nmol/l) and hemodynamic parameters (CO 3.2 l/minute; SVR 550 dyne seconds/cm^5^, MAP 70 mmHg). Her body temperature was 36.5 °C. GCS score remained 5 (E1, V1T, M3) when another sedation window was performed. Her hemodynamic stability allowed her to undergo a brain magnetic resonance imaging (MRI) scan (Fig. 1). The MRI scan revealed an altered signal and post-contrast enhancement of the leptomeninges. Moreover, multiple T2 and fluid-attenuated inversion recovery (FLAIR) hyperintense and sometimes confluent lesions were detected: in the thalamus, which appeared swollen; in the pons, in the cerebellar peduncles, and in cerebellar hemispheres, also appearing swollen; in the ventricles (mostly in the lateral ventricles and in the occipital horn bilaterally); and in the parahippocampal region bilaterally. All these lesions were also characterized by a reduced diffusivity in diffusion-weighted imaging (DWI) scans.

**Fig. 1.**
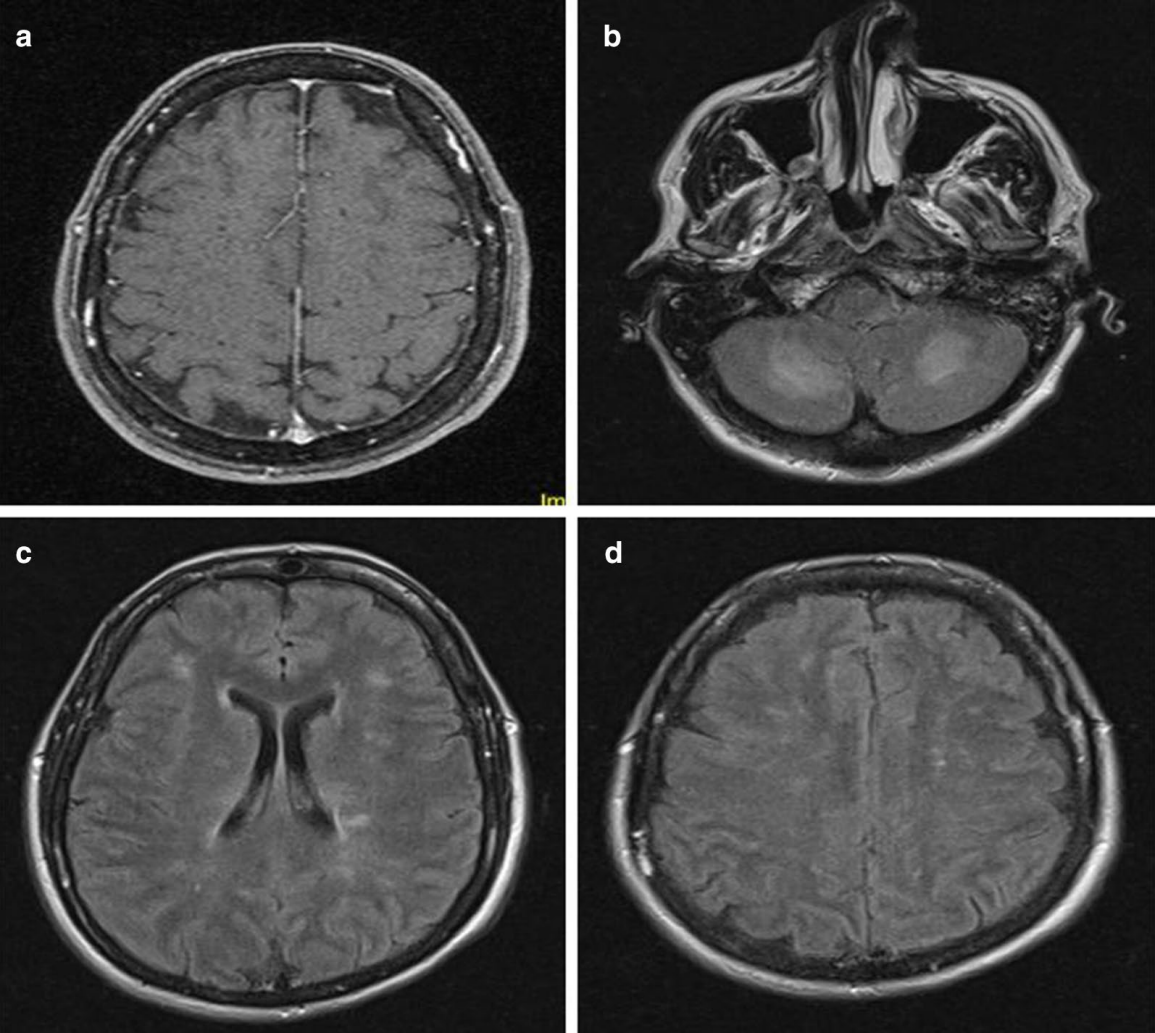
Images from the first MRI scan. **a** T1-weighted image showing meningeal post-contrast enhancement. **b** FLAIR image demonstrating bilateral cerebellar hyperintense lesions in both hemispheres. **c** FLAIR image showing periventricular hyperintensity and multiple white-matter hyperintense lesions. **d** FLAIR image demonstrating multiple juxtacortical white-matter lesions

Finally, multiple comminute T2/FLAIR white-matter hyperintense lesions were demonstrated, located in the juxtacortical white matter, especially in frontal regions, in both the corona radiata, and in periventricular regions bilaterally.

After 48 hours from the start of Pentaglobin treatment, there was a remarkable improvement in hematochemical and hemodynamic parameters. In particular, the patient no longer needed inotropic support, and we therefore suspended continuous infusion of norepinephrine. Ventilation was switched to an assisted mode to wean the patient from the ventilator. Three days after admission, hemodynamic parameters of the patient were still improving, she was not febrile anymore, and lactate levels were dropping; on the other hand, her neurological condition was still severe, with a persistent altered mental state, bilateral miosis with a torpid papillary response, nystagmus, dyplegia with bilaterally positive Babinski sign and hyperelicitable osteotendinous reflexes.

96 hours after Pentagoblin introduction, there was an evident improvement in the patient's clinical condition. GCS score increased to 10 (E3, V1T, M6). The patient was also able to be extubated, breathing spontaneously. Blood chemistry values were CPR 5.18 mg/dl, PCT 1.2 ng/ml, and lactate 1.2 mmol/l. Hemodynamic values were CO 5.4 l/minute, SVR 1200 dynes second/cm^5^, MAP 90 mmHg final. Neurological examination showed normal pupils and pupillary response, dyplegia with bilaterally positive Babinski sign, and hyperelicitable osteotendinous reflexes. Other cerebellar signs besides nystagmus became evident, with dysarthria and dysmetria of the upper limbs. A mild cognitive impairment was also detected, as the patient showed apraxia and executive functioning deficits. As the patient was more responsive, both a physiatrist and speech therapist assessment were scheduled to evaluate her and commence rehabilitation.

Six days after admission, the patient underwent a control CT scan, which demonstrated a partial resolution of the renal alterations suggestive of pyelonephritis. She also underwent a control brain MRI on day 9 after admission. The MRI scan showed a reduction of all the previously detected lesions, with less swelling of the thalamus and cerebellum. On the other hand, the multiple juxtacortical and periventricular white-matter lesions remained substantially unchanged, also showing some microbleeding spots (Fig. 2)

**Fig. 2.**
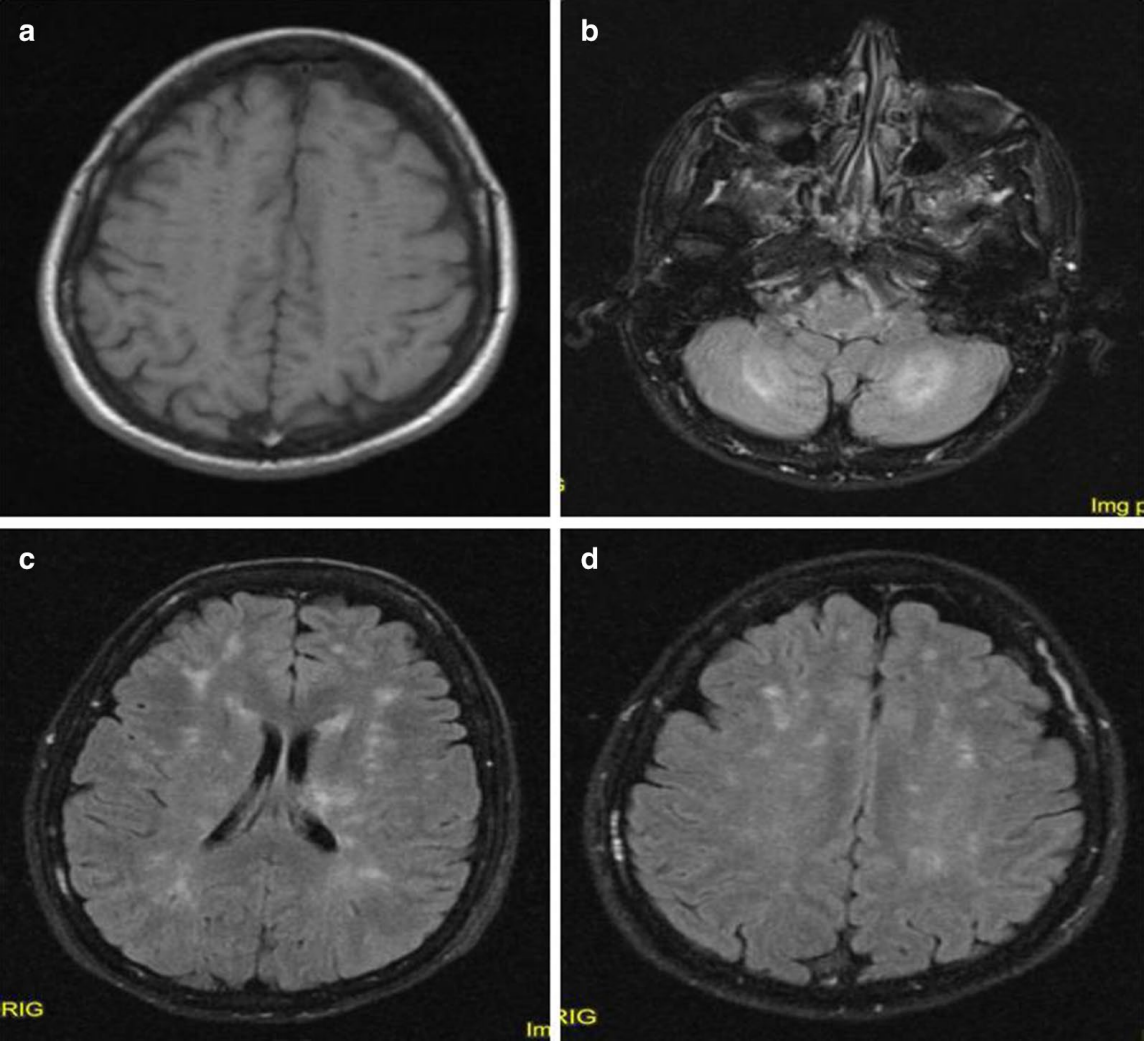
Images from the control MRI scan. **a** T1-weighted image without meningeal post-contrast enhancement. **b** FLAIR image demonstrating a reduction of hyperintense lesions in both cerebellar hemispheres. **c**, **d** FLAIR images still showing a significant bulk of hyperintense lesions involving periventricular, deep, and juxtacortical white matter

The patient remained in our department for the continuation of antimicrobial therapy and close monitoring. Twenty-one days after admission the patient was remarkably improved, showing only mild cerebellar signs (mostly dysarthria, along with dysmetria and a slight action tremor), slight hyposthenia of the four limbs, and mild apraxia on neurological examination. She was discharged and transferred to a rehabilitation center for post-intensive care rehabilitation to regain limb strength and coordination and to improve her speech abilities. Physiatrist, speech therapy, and neurological out-patient consultations, as well as a 6-month control brain MRI scan, were scheduled. Three months after discharge, a striking improvement of her condition was reported, as she was almost free from any neurological sign or symptom and almost fully recovered from her condition.

## Discussion and conclusion

We reported what we consider to be a peculiar case report because of several reasons: first of all, from an epidemiological and clinical point of view. Gram-negative bacteria and in particular *Escherichia coli* strains are a rare cause of spontaneous community-acquired Meningitis in adults [4, 5]. *Escherichia coli* strains are a relevant etiology of meningitis and meningoencephalitis in neonates and infants [13, 14], being a relevant cause of both mortality and disability in this cohort of young patients [15, 16]. In adults *E. coli* is, not unlike other Gram-negative bacteria, more often responsible for nosocomial meningitis/meningoencephalitis, in particular in posttraumatic and/or postneurosurgical patients [6–8, 17–19]. On the other hand, as mentioned above, *Escherichia coli* accounts only for 1–3% of spontaneous community-acquired adult meningitis/meningoencephalitis [5–8]. Moreover, according to recent reviews of the few described cases of *E. coli* meningitis or meningoencephalitis [6, 7], many of the patients had at least one relevant comorbidity or risk factor, such as cirrhosis or chronic alcoholism, diabetes mellitus, a history of chronic organ dysfunction, HIV infection, or another cause of immunodeficiency such as prolonged corticosteroid therapy or cancer. Conversely, our patient had no history of relevant comorbid conditions. Also, a primary distant focus of infection is often detected, such as urinary tract infections (UTIs), pneumonia, septic arthritis, otitis media, and peritonitis [5–7, 20]. Our patient indeed had both radiological and microbiological evidence of a UTI, which is the most frequently reported associated infection. Regardless of the source of infection, meningeal and encephalic involvement appear to be strongly connected to bacteremia, and in particular with a high degree of bacteremia [5–7, 21]. Given that the patient arrived with a septic shock condition, a high degree of bacteremia was likely present. Beyond the degree of bacteremia, other bacterial virulence factors such as the K1 capsule or fimbrial and flagellar proteins appear to be implicated in *E. coli* transmigration through the blood–brain barrier (BBB) by binding and invasion of human brain microvascular endothelial cells (HBMEC) [21]. This subgroup of *Escherichia coli* is often defined as “extraintestinal pathogenic *Escherichia coli*” (ExPEC) and comprises strains that are neither commensal in human gut nor responsible for isolated gastrointestinal infections. The ExPEC group includes uropathogenic *E. coli* (UPEC), neonatal meningitis *E. coli* (NMEC), sepsis-associated *E. coli* (SEPEC), and avian pathogenic *E. coli* (APEC); all of these have several virulence factors, including adhesins, toxins, and invasins [22].

In general, risk factors for adult meningitis mortality are reported to be advanced age, female sex, altered mental state/coma, hypotension, and seizures [5, 23]. Overall reported mortality for *E. coli* adult meningitis ranges between 47% and 90%; the rate of neurological sequelae among survivors, including focal signs and cognitive impairment, is about 30–50% [5–8]. Our patient indeed had a complicated presentation: systemic impairment with sepsis and septic shock, a severe degree of mental status alteration with coma, and multiple focal neurological signs (including dyplegia with a bilaterally positive Babinski sign, and cerebellar impairment). Moreover, multiple brain lesions beyond leptomeningeal enhancement were detected on MRI examination, thus indicating meningoencephalitis. Indeed, a certain degree of brain inflammation is unavoidable in bacterial meningitis, because of both pathogen toxicity factors [21, 22, 24, 25] and host response to infective aggression. Several host-related causes of neuronal damage have been investigated, including leukocytary and microglial activation, cytokine and chemokine release, neurotransmitter dysfunction, and oxidative damage; the imbalance between an exaggerated physiological immune response and the regulatory antiinflammatory immune pathways ultimately leads to neuronal damage and death. Brain edema and secondary vasculitis may also be responsible for ischemia and further neuronal damage [26–30]. Some of these neuroinflammatory mechanisms are also shared with septic encephalopathy [31–33]; although this condition should occur without evidence of an intracranial infection, our patient did demonstrate systemic involvement as she was in a state of septic shock. Therefore, besides direct damage following meningeal aggression, other septic encephalopathy pathophysiological mechanisms, such as microglial activation, neurotransmitter imbalance, oxidative/mitochondrial dysfunction, and macrovascular and microvascular/endothelial impairment [31–33] might have played a role in our patient’s condition.

MRI examination is another peculiar issue of our case that is worth highlighting. MRI allows the detection of subtle changes in brain and meningeal parenchyma, helps in differential diagnosis, and is a useful tool to monitor complications, treatment response, and disease evolution [34–38]. Meningitis may be accompanied by several other alterations that are reported in a considerable number of patients: ventricular involvement, with ependymal enhancement in T1 scans, hyperintense images in T2/FLAIR imaging, and reduced diffusivity in DWI; hydrocephalus; cerebral edema; cerebritis with T2/FLAIR hyperintense lesions and a reduction of diffusion in DWI, mostly involving cortical regions and juxtacortical white matter; vascular congestion, venous thrombosis, and ischemic lesions; and subdural empyema and cerebral abscesses [34–38]. Our patient did display some of these alterations, such as leptomeningeal enhancement, ventricular/ependymal involvement, multiple T2/FLAIR hyperintense lesions, and multiple areas of diffusivity reduction in DWI scans. Of note, she had dramatic cerebellar involvement; cerebellitis is a rare condition in adult patients, being more common in pediatric populations [39, 40]. Moreover, although bacterial etiology is seldom reported, cerebellar inflammation is more often caused by viral infection or postinfection and autoimmune conditions [39, 40]. Although a full recovery is infrequently described in adult patients [39], she exhibited a brilliant recovery from her cerebellar symptoms in only a few weeks.

Lastly, we would like to emphasize the adjuvant role of IgM-enriched Immunoglobulin in treating the severe condition of our patient. As mentioned above, the Surviving Sepsis Campaign (SSC) guidelines advise against the use of IVIG preparations in patients with sepsis or septic shock [10]. Nevertheless, several considerations supported our decision to administer IgM-enriched IVIGs early in the treatment schedule of our patient: first of all, the relatively young age and the dramatic presentation on admission, with a state of severe septic shock. Moreover, the SSC panel itself rates the guideline as a “weak recommendation, low quality of evidence” [10]. Indeed, different studies have addressed the utility of IVIGs and IgM-enriched IVIGs in patients with sepsis or septic shock, and some reviews and meta-analyses have investigated the results of these studies. The main investigated outcome was survival; also, influence of the treatment on the need of mechanical ventilation, length of stay (LOS) in the ICU, Sequential Organ Failure Assessment (SOFA) score, vasopressor use, and serum analytes such as IL-6 or endotoxin were assessed [12, 41–46] as secondary outcomes. Regarding mortality, conflicting results are reported, as some studies underscored an improvement in survival rates in treated patients, whereas other investigations demonstrated no statistically significant benefit for IVIG use [12, 41–47]. This may depend on several reasons: first of all, only a few studies are randomized clinical trials (retrospective and cohort designs have also been performed), and some of the analyzed studies have been published before the 2000s, which might influence patient selection as definitions of sepsis and septic shock have been modified over the years. Moreover, there is a substantial heterogeneity in control interventions (albumin or placebo), dosage and duration of administration, and IVIG formulations in the different studies [12, 41–47]. Nevertheless, IgM-enriched IVIG appears to benefit survival rate, although further investigations are surely needed to better disclose the real impact of IgM-enriched IVIG on mortality. In addition, a reduction in the need for mechanical ventilation and a drop in endotoxin activity have been observed [12, 41]. IVIGs are undoubtedly an intriguing adjuvant therapy in severely affected patients, as several mechanisms of action, such as modulation of immune cell functions such as cytokine production and response or killing and autophagy activity of neutrophils, support their utility in sepsis or septic shock patients [12, 46–48].

Lastly, a recent study reported higher efficacy of IVIG preparations on survival outcomes when administered early in the course of sepsis and septic shock [49]. Therefore, we also decided to introduce IgM-enriched IVIGs early after admission.

Gram-negative bacteria , in particular *E. coli*, are uncommon etiologies in spontaneous community-acquired bacterial meningitis; nevertheless, prompt diagnosis and therapy are crucial to enhance the chances of survival and reduce the odds of permanent sequelae. Timely administration of an aggressive and targeted antimicrobial therapy along with an adjuvant therapy with corticosteroids and IgM-enriched IVIGs achieved a remarkable resolution of both meningoencephalitis and septic shock. In spite of a relevant risk of mortality and permanent neurological disability giving the infection’s etiology and the dramatic presentation on admission, our patient experienced a brilliant recovery with no deficits and almost complete resolution of neurological signs and symptoms.

## Data Availability

The datasets used and analyzed during the current study are available from the corresponding author on reasonable request.

## Abbreviations

ExPEC: Extraintestinal pathogenic *Escherichia coli*
ER: Emergency room
ICU: Intensive care unit
CT: Computerized tomography
CO: Cardiac output
SVR: Systemic vascular resistance
PCT: Procalcitonin
SpO_2_: Peripheral saturation of O_2_
MAP: Mean arterial pressure
GCS: Glasgow Coma Scale
WBC: White blood cells
RBC: Red blood cells
HB: Hemoglobin
PLT: Platelets
CPR: C-reactive protein
CFU: Colony-forming units
IVIG: IgM-enriched intravenous immunoglobulin
MRI: Brain magnetic resonance imaging
FLAIR: Fluid-attenuated inversion recovery
DWI: Diffusion-weighted imaging
BBB: Blood–brain barrier
HBMEC: Human brain microvascular endothelial cells
UPEC: Uropathogenic *E. coli*
NMEC: Neonatal meningitis *E. coli*
SEPEC: Sepsis-associated *E. coli*
APEC: Avian pathogenic *E. coli*
DIC: Disseminated intravascular coagulation
